# Time-Dependent Shrinkage Model for Recycled Fine Aggregate Thermal Insulation Concrete

**DOI:** 10.3390/ma14195581

**Published:** 2021-09-26

**Authors:** Xuhang Zang, Pinghua Zhu, Chunhong Chen, Xiancui Yan, Xinjie Wang

**Affiliations:** Changzhou City Key Laboratory of Building Energy-Saving Technology, Department of Civil Engineering, Changzhou University, Changzhou 213164, China; zangxuhangcczu@163.com (X.Z.); chench@cczu.edu.cn (C.C.); hhuyanxc@163.com (X.Y.); wangxinjie@cczu.edu.cn (X.W.)

**Keywords:** glazed hollow bead, recycled fine aggregate, recycled fine aggregate thermal insulation concrete, time-dependent shrinkage model, shrinkage development

## Abstract

In this study, the shrinkage performance of recycled aggregate thermal insulation concrete (RATIC) with added glazed hollow beads (GHB) was investigated and a time-dependent shrinkage model was proposed. Two types of recycled fine aggregate (RFA) were used to replace natural fine aggregate in RATIC: RFA from waste concrete (RFA1) and waste clay brick (RFA2). Besides, the mechanical properties and thermal insulation performance of RATIC were also studied. Results showed that the pozzolanic reaction caused by RFA2 effectively improved the mechanical properties of RATIC; 75% was the optimal replacement ratio of RATIC prepared by RFA2. Added RFA decreased the thermal conductivity of thermal insulation concrete (TIC). The total shrinkage strain of RATIC increased with the increase of the replacement ratio of RFA. The 150d total shrinkage of RATIC prepared by RFA1 was 1.46 times that of TIC and the 150d total shrinkage of RATIC prepared by RFA2 was 1.23 times. The addition of GHBs led to the increase of early total shrinkage strain of concrete. Under the combined action of the higher elastic modulus of RFA2 and the pozzolanic components contained in RFA2, the total shrinkage strain of RATIC prepared by RFA2 with the same replacement ratio was smaller than that of RATIC prepared by RFA1. For example, the final total shrinkage strain of RATIC prepared by RFA2 at 100% replacement ratio was about 18.6% less than that of RATIC prepared by RFA1. A time-dependent shrinkage model considering the influence of the elastic modulus of RFA and the addition of GHB on the total shrinkage of RATIC was proposed and validated by the experimental results.

## 1. Introduction

With the development of concrete construction, a large number of old buildings with brick and concrete structures will be demolished. Recycled coarse aggregate (RCA) and recycled fine aggregate (RFA) are the main products after the demolishment of brick and concrete structures [1]. For the RFA, recycled fine aggregate of waste concrete (RFA1) and recycled fine aggregate of waste clay brick (RFA2) are two common types [2]. Recycling RFA can effectively solve the shortage of natural river sand resources. Recently, the mechanical properties and durability of concrete containing RFA1 and RFA2 have been studied. Wang et al. [3] found that the compressive strength of recycled fine aggregate concrete (RFAC) containing RFA1 at 28 days was greater than the ordinary concrete. Kirthika et al. [4] systematically studied the durability of RFAC with RFA1 and results showed that concrete with 30% RFA1 had better resistance to chloride penetration and carbonation. Dang et al. [5] found that the compressive strength of RFAC containing 50% RFA2 with no extra water was comparable with ordinary concrete. It is a significant issue to study the performance of RFAC for the utilization of RFA.

Recycled aggregate thermal insulation concrete (RATIC) is a new type of green structural concrete, with the glazed hollow beads (GHB) and recycled aggregate [6,7]. It not only alleviates environmental problems caused by construction and demolition waste but also reduces the overall energy consumption of buildings [8]. At present, research mainly focuses on the mechanical properties and thermal conductivity of RATIC. Wang et al. [9] carried out a systematic study on the mechanical properties of RATIC prepared with RCA, and the results showed that the compressive strength, splitting tensile strength and elastic modulus of RATIC were all lower than those of the GHB thermal insulation concrete (TIC). Xiao et al. [10] found that the thermal conductivity of recycled concrete decreased with the increase of the replacement ratio of RCA. The test results of Zhao et al. [11] showed that the thermal conductivity of concrete decreased significantly with the increase of GHB content. Nevertheless, few reports related to the performance of RATIC containing RFA.

As a new green structural concrete, the shrinkage of concrete causes micro-cracks [12] and plays an important role in the design of the service limit state of structural members [13]. According to EN1992 [14], the total shrinkage of concrete is composed of two parts: autogenous shrinkage and drying shrinkage of concrete. Autogenous shrinkage of concrete is defined as the volume reduction that occurs without moisture exchange between the internal concrete and external environment [15]. Delsaute et al. [16] found that RFA1 was beneficial to reducing the autogenous shrinkage of concrete, which attributed to the combined effect of additional water and elastic modulus of RFA1. Zhang et al. [17] found there was a rapid increase in the autogenous shrinkage of RFAC during the first 60 days, and then the increasing rate reduced. Moreover, Zhang et al. [18] find that the autogenous shrinkage strain of RFAC prepared by RFA2 decreased with the increase of the RFA2 replacements. Drying shrinkage is the volume reduction of hardened concrete caused by evaporation of water under the effect of unsaturated environmental humidity [19]. Khatib et al. [20] made a comparative study on the drying shrinkage of RFAC prepared by RFA1 and RFA2, and the 90-day drying shrinkage of RFAC prepared by RFA2 was significantly smaller than that prepared by RFA1. Zhang et al. [21] selected two different types of RFA1 and found that the water absorption and elastic modulus of RFA1 were the main reasons for the large difference in the final drying shrinkage strain of RFAC; based on the results, Zhang et al. [21] introduced the correction coefficients *k_wa_* and *k_v_* to modify the drying shrinkage model of ordinary concrete in EN1992 [14], which considered the influence of water absorption on the drying shrinkage development and the influence of elastic modulus on the final drying shrinkage strain of RFA1, respectively. Dang et al. [1] attributed the increase of drying shrinkage of RFAC prepared by RFA2 to the low elastic modulus of RFA2. Although many researches on the shrinkage of RFAC have been conducted, little information on the shrinkage of RATIC can be found in the literature.

At present, it is not common to conduct long-term shrinkage test as a routine test for every single concrete mixture, the structural engineer can only assess the risk of concrete cracking using existing prediction models [22]. In order to better represent the shrinkage of concrete in precast components, a large number of autogenous shrinkage, drying shrinkage and total shrinkage models have been established [23]. Maghfouri et al. [22] used five existing concrete shrinkage models to compare the shrinkage strain behavior of light weight concrete containing coarse aggregate and found that the EC2 model was the best prediction model to simulate the early shrinkage of light weight aggregate concrete. Bunderen et al. [24] compared the error between the total shrinkage model and the drying shrinkage model of concrete, and found that EC2 model could also be used to predict the drying shrinkage of concrete. Lai [25] et al. considered the influence of cementitious paste volume and wet packing density on the total shrinkage of concrete, and reduced the root mean square error of total shrinkage by optimizing the drying shrinkage in the AS3600 shrinkage equation. However, the prediction model of total shrinkage of RATIC containing GHB cannot be found in the literature.

In the present study, the shrinkage performance of RATIC containing GHB was investigated. Considering the influence of RFA and GHB, a time-dependent shrinkage model was proposed to predict the total shrinkage of RATIC. Besides, the mechanical properties, thermal insulation performance and the scanning electron microscope (SEM) of RATIC were also studied.

## 2. Materials and Methods

### 2.1. Cementing Material

P•O 42.5 cement and fly ash were used as cementitious materials. The chemical components of cementitious materials listed in Table 1 met the requirements of Chinese standard GB/T 175-2007 [26].

### 2.2. Aggregates

**Figure 1:**
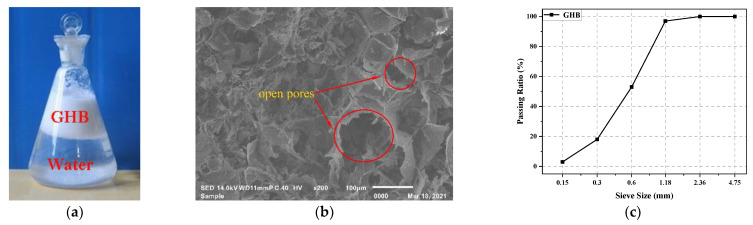
(**a**) The GHBs after soaking in water for three months and (**b**) SEM image of GHB and (**c**) Sieve size distribution of GHB.

GHBs, produced by Jiangsu Huajia Building Materials Technology Co., Ltd, Zhenjiang, China, was added to concrete as a fine aggregate. The physical properties of GHBs are listed in Table 2. Figure 1a shows the GHBs after soaking in water for three months. The higher flotation ratio of GHBs in Figure 1a indicated that it was difficult for water to enter the inner cavity of GHB, and the absorbed water of GHBs was mainly attached to the surface or stored in the open pores. Figure 1b is the SEM image of GHB, open pores can be seen on the surface of GHB. Figure 1c is the sieve size distribution of GHB.

Gravel and river sand was used as natural coarse aggregate (NCA) and NFA, respectively. Two different types of RFA were also used. RFA1 was prepared from waste concrete and RFA2 was prepared from waste clay bricks. The physical properties of aggregates were tested according to the Chinese standard GB/T 14684–2011 [28] and GB/T 14685–2011 [29], as shown in Table 3. Among them, the water absorption, moisture content and crushing value of RFA1 were higher than that of RFA2, and the apparent density was lower than that of RFA2. Overall, the physical properties of RFA1 were worse than that of RFA2.

**Figure 2:**
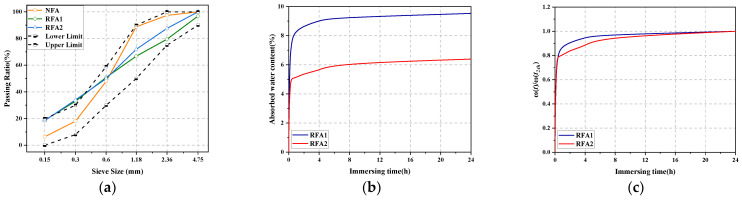
(**a**) Sieve size distribution of fine aggregates and (**b**) the variation of water content with time in RFA and (**c**) the variation trend of RFA water absorption after normalization treatment.

The particle size of coarse aggregate was 5–20 mm. In order to increase the performance comparability of different types of concrete, all gradations of fine aggregates were adjusted to make RFA1 and RFA2 gradations as consistent as possible with NFA gradations. The particle size distribution of the three kinds of fine aggregate is shown in Figure 2a, and their fineness modulus was all 2.4. The volumetric flask method was used to test the 24-h water absorption rate of RFA [30]. Figure 2b shows the variation of water content with time in RFA, and Figure 2c shows the variation trend after normalization treatment [31], that is the calculated value of *ω*(*t*)/*ω*(*t*_24h_). As can be seen from Figure 2c, the water absorption rate of RFA1 was faster than that of RFA2.

**Figure 3:**
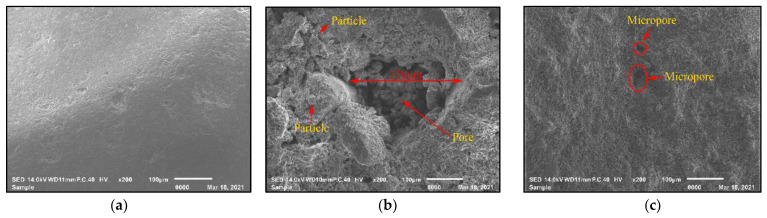
SEM images of (**a**) NFA, (**b**) RFA1 and (**c**) RFA2.

Figure 3a–c is the SEM images of NFA, RFA1 and RFA2, respectively. The surface of NFA was smooth and flat (Figure 3a), while there were obvious pores on the surface of RFA1(Figure 3b). In addition, many small particles occurred on the surface of RFA1, namely old mortar particles in waste concrete. There were distinct lines on the surface of RFA2 (Figure 3c), which should be the sintered phase produced in the sintering process of clay bricks. RFA2 had a compact surface and clear structure with only a few micro pores. Comparing Figure 3b,c shows that the surface of RFA2 was denser than that of RFA1, indicating a better quality of RFA2.

**Figure 4:**
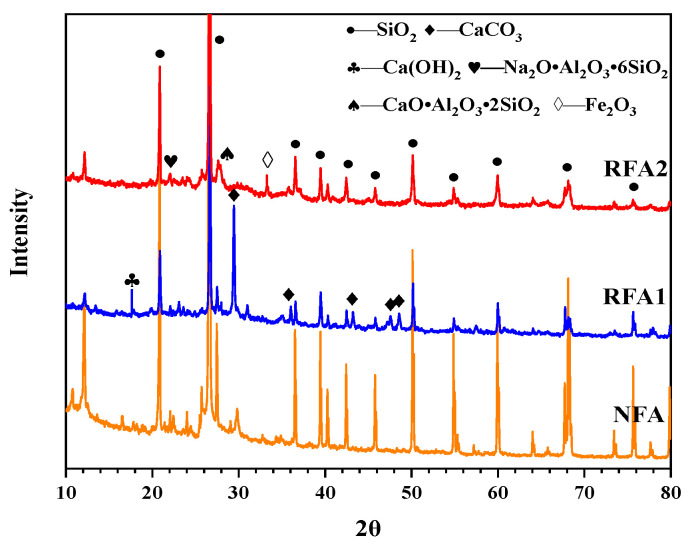
X-ray diffraction diagrams of fine aggregate.

The chemical composition and main oxides of fine aggregate were analyzed by X-ray powder diffractometer (Rigaku, Japan) and X-ray fluorescence spectrometer (Bruker, Germany), respectively. The main oxides in fine aggregate are listed in Table 4. The chemical composition of fine aggregate is shown in Figure 4. It can be seen from Table 4 that the main chemical composition of NFA was SiO_2_, while the chemical composition of RFA1 and RFA2 were more complex. The content of CaO in RFA1 was as high as 27.91%. It can be seen from the XRD of RFA1 in Figure 4 that the main existing forms of CaO in RFA were CaCO_3_ and Ca(OH)_2_. Compared with RFA1, the contents of Al_2_O_3_ and Fe_2_O_3_ in RFA2 were 6.35% and 3.74% higher, respectively. The total content of SiO_2_, Al_2_O_3_ and Fe_2_O_3_ in RFA2 was more than 70%, which met the requirement of the content of main chemical components of pozzolanic materials in ASTM C618 [32]. Therefore, RFA2 with sieve size less than 0.15 mm could be considered as a potential pozzolanic material [33].

### 2.3. Mix Proportions

In this study, RFA1 and RFA2 were used to replace NFA with different replacement ratios (25%, 50%, 75% and 100%) to prepare recycled aggregate thermal insulation concrete, namely RATIC1 and RATIC2. Considering the influence of GHBs and RFAs on the shrinkage performance of concrete, a group of NAC and a group of TIC specimens were designed as the control groups. Mix proportions of different types of concrete are shown in Table 5. In order to improve the performance of RATIC [34,35], additional water was added in the stirring process, where the additional water consumption was obtained by multiplying the difference between the 24-h water absorption and the moisture content of RFA by the mass amount of RFA. The superplasticizer with solid content of 40% was used to improve the performance of concrete.

### 2.4. Specimen Preparation

**Figure 5:**
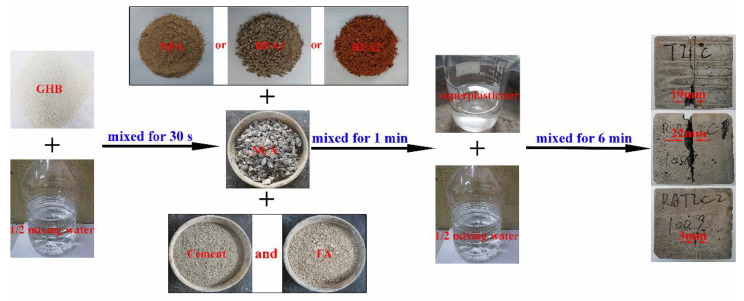
Schematic view of mixing procedures.

The concrete mixing method referred to the method proposed by Wang et al. [6], as shown in Figure 5. For each type of concrete mixture, twenty-four 100 × 100 × 100 mm cubes, three 150 × 150 × 300 mm prisms, three 100 × 100 × 515 mm prisms and two *ϕ*200 × 400 mm cylinders were prepared.

### 2.5. Test Methods

The slump of mixed concrete was measured according to GB/T 50080-2016 [36]. After casting, all specimens are maintained according to the Chinese standard [37]. The compressive strength of concrete at 3, 7, 14, 28, 60, 90, 150 days and the splitting tensile strength at 28 days were tested using the 100 × 100 × 100 mm cubes. The elastic modulus of concrete at 28 days was evaluated using the 150 × 150 × 300 mm prisms. The thermal conductivity of concrete at 28 days was evaluated using the *ϕ*200 × 400 mm cylinders.

**Figure 6:**
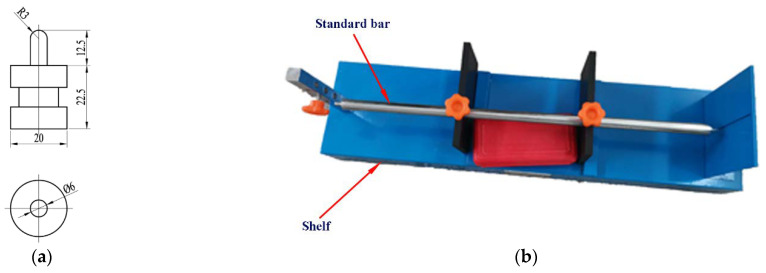
(**a**) Size of the probe and (**b**) HSP-540 concrete shrinkage dilatometer.

The shrinkage performance of concrete was tested according to GB/T 50082-2009 [38]. The shrinkage of concrete was measured using the 100 × 100 × 515 mm prisms. First, the specimen was put into standard curing room for 3 days; then, the specimen was immediately moved into a test chamber where the room temperature was maintained at (20 ± 2) °C and the relative humidity was maintained at (60 ± 5) %. HSP-540 concrete shrinkage dilatometer (Blue standard building instrument Factory, Cangzhou, Heibei, China) was used to test the length of RATIC at different times, and the total shrinkage strain value of RATIC was obtained. The embedded probe was used in this test, and the size of the probe and HSP-540 concrete shrinkage dilatometer is shown in Figure 6. The initial calibration distance of the specimen was 470 mm, and the total shrinkage rate of concrete was measured over a period of 150 days.

The microstructure of the ITZ of concrete between GHB and cement matrix, the ITZ of concrete between fine aggregate and cement matrix were observed by SEM. Before the experiment, the samples were soaked in ethanol for 24h, and then dried in a vacuum-desiccant box at 60 °C for 24 h. The SEM images were captured by an JSM-IT100 equipment (JEOL, Japan) at 14 kV after the samples were spray-coated with gold.

## 3. Results and Discussion

### 3.1. Mechanical Properties of RATIC

#### 3.1.1. Compressive Strength (f_c_)

**Figure 7:**
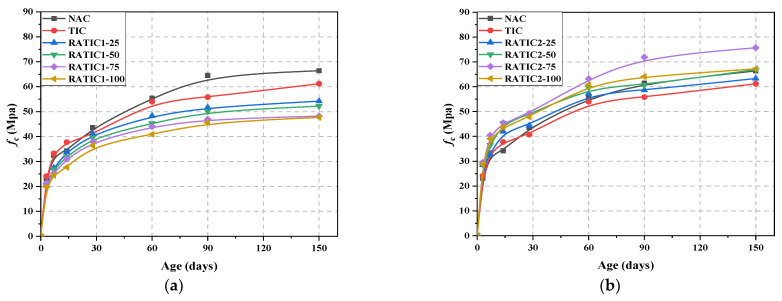
The compressive strength of RATIC at different ages: (**a**) RATIC1; (**b**) RATIC2.

The compressive strength of RATIC at different ages is shown in Figure 7. Generally, the compressive strength of RATIC increased with the increasing ages. At the early ages, the difference between the compressive strength of NAC and TIC was narrow; adding GHB had no obvious effect on the compressive strength of TIC before 28 days. However, the compressive strength of NAC after 28 days was a little higher than that of TIC. At 150 days, the compressive strength of NAC was 8.5% higher than that of TIC.

As can be seen in Figure 7a, the compressive strength of RATIC1 decreased with the increase of replacement ratio of RFA1. For example, the compressive strength of RATIC containing 100% RFA1 at 150 days decreased by 22.1% compared to that of TIC containing no RFA1. However, in Figure 7b, the compressive strength of RATIC increased when RFA2 added. The maximum compressive strength of RATIC2 occurred in the RATIC2 specimen containing 75% RFA2. The compressive strength of RATIC2-75 at 150 days increased by 23.7% compared to that of TIC. 

The chemical composition and physical properties of RFA1 and RFA2 were important factors affecting the compressive strength of RATIC1 and RATIC2. RFA1 had a higher water absorption and absorbed a large amount of water, which slowed down the hydration rate of cement. Besides, the crushing value of RFA1 was higher than that of RFA2. The poor physical and mechanical properties of RFA1 had a negative effect on the compressive strength of RATIC. For the RATIC2, the water absorption of RFA2 was smaller than that of RFA1. Adding RFA2 increased the compressive strength of RATIC, which might be due to the reaction between Ca(OH)_2_ and pozzolanic material, producing new hydrated calcium silicate and providing additional strength for RATIC2.

#### 3.1.2. Splitting Tensile Strength (f_spt_)

The splitting tensile strength of RATIC at 28 days is shown in Table 6. Generally, the splitting tensile strength increased first and decreased later with the increasing of RFA contents. For the RATIC1, the maximum splitting tensile strength at 28 days occurred in the specimen containing 50% RFA1; for the RATIC2, the maximum splitting tensile strength occurred in the specimen containing 75% RFA2. When the replacement ratio of RFA was relative low, the combination of the irregular surface of RFA and the penetration of the cement matrix to the surface of the RFA contributed to enhance the adhesion force of ITZ and improved the mechanical properties of RATIC [5]. When the RFA contents increased, the larger crushing value of RFA could exert a bad effect on the mechanical properties of RATIC. In addition, it was found that the splitting tensile strength of NAC at 28 days was slightly larger than that of TIC, which is in accordance with the results of compressive strength.

#### 3.1.3. Elastic Modulus (E_c_)

The elastic modulus of RATIC at 28 days is shown in Table 7. It can be seen that adding GHB greatly decreased the elastic modulus of RATIC. The elastic modulus of TIC at 28 days was nearly 26.4% less than that of NAC, which could be owing to the porous property of lightweight aggregate of GHB. This phenomenon was similar with the results in [11] that adding GHB greatly reduced the elastic modulus of ordinary concrete. In addition, the variation of elastic modulus of RATIC was similar with the splitting tensile strength; it increased first and decreased later with the increasing of RFA contents. The maximum elastic modulus of RATIC1 and RATIC2 occurred in specimen with 50% RFA1 and 75% RFA2, respectively. Besides, the elastic modulus of RATIC2 were higher than that of RATIC1. It can be seen from Table 3 that RFA2 has better physical properties, which was beneficial to the mechanical properties of RATIC. Besides, the pozzolanic composition in RFA2 can also improve the elastic modulus of RATIC2 [33].

### 3.2. Thermal Conductivity (K_c_)

The thermal conductivity of RATIC at 28 days is shown in Table 8. In Table 8, the thermal conductivity of NAC reduced nearly 40.1% when GHB added, indicating that TIC had a better thermal insulation performance than NAC. In addition, the thermal conductivity of TIC could decrease when RFA added. For the RATIC1, the minimum thermal conductivity occurred in specimen with 100% RFA1; for the RATIC2, the minimum thermal conductivity occurred in specimen with 100% RFA2. This phenomenon might be attributed to the pores on the surface of the RFA that were not fully filled, which reduced the thermal conductivity of the RATIC. Besides, irrespective of the RFA contents, the thermal conductivity of RATIC1 was slightly smaller than that of RATIC2, which could be due to the more and larger pores in RFA1.

### 3.3. Total Shrinkage (ε_cs_)

**Figure 8:**
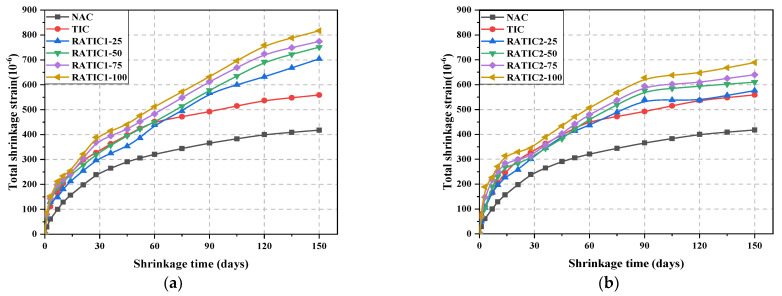
The total shrinkage curve of RATIC with time: (**a**) RATIC1; (**b**) RATIC2.

The total shrinkage curve of concrete is shown in Figure 8. In Figure 8, the total shrinkage strain of TIC was greater than that of NAC. This may be because the addition of GHB reduced the overall elastic modulus of concrete aggregate and increased the air content of concrete. The decrease of the elastic modulus of the aggregate reflected the decrease of compressive strength of concrete to some extent. Therefore, it was no surprise that the final total shrinkage strain of TIC was larger than that of NAC.

In addition, the total shrinkage strain of RATIC increased with the increase of RFA replacement ratio. The total shrinkage strain of RATIC1-100 at 150 days was 1.46 times that of TIC, and the total shrinkage strain of RATIC2-100 at 150 days was 1.23 times that of TIC. Besides, comparing Figure 8a,b shows that the total shrinkage strain of RATIC1 was larger than RATIC2, irrespective of the contents of RFA. This phenomenon could be due to the lower elastic modulus of RATIC1; the lower elastic modulus of aggregate led to a higher shrinkage [21].

**Figure 9:**
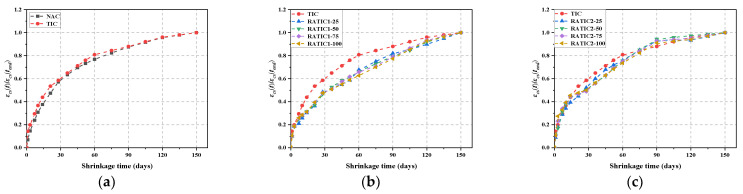
Normalized total shrinkage of (**a**) NAC and TIC, (**b**) RATIC1 and (**c**) RATIC2.

In order to compare the development of total shrinkage of RATIC, the total shrinkage strain of NAC, TIC and RATIC at different times was normalized to the final value, that is, *ε_cs_* (*t*)/ *ε_cs_* (*t_end_*), as shown in Figure 9a–c. As can be seen from Figure 9a, compared with NAC, the total shrinkage strain of TIC developed rapidly in the early stage, and the total shrinkage at 1 day and 3 days reached 14.31% and 19.86% of the total shrinkage, respectively, which were 106.3% and 36.1% higher than the corresponding total shrinkage of NAC. The main reason for this phenomenon may be the continuous progress of cement hydration, which had a compaction effect on GHBs [39]; the volume deformation of GHBs occurred to a certain extent, which could increase the total shrinkage strain of RATIC at early stage. Maghfouri et al. [22] reached similar conclusions by adding oil palm shells to concrete. As also shown in Figure 9a, GHBs had little effect on the long-term development of total shrinkage strain of TIC, which may be due to the fact that the water adsorbed on the surface of GHBs and the water in the open pores dissipated quickly.

As can be seen from Figure 9b,c, the total shrinkage strains of RATIC1-100 at 3, 7 and 14 days were 18.7%, 25.9% and 31.2% of their 150 d total shrinkage strains, respectively. The total shrinkage strains of RATIC2-100 at 3, 7 and 14 days were 27.4%, 32.9% and 45.6% of their 150 d total shrinkage strains, respectively. In contrast to RATIC1, in the early stages of the total shrinkage development of RATIC2, the water was not only used for the cement hydration reaction, but also provided the water needed for the pozzolanic reaction in RFA2. Therefore, the internal environment of RATIC2 was drying faster than that of RATIC1 [40], which might be the main reason for the rapid development of total shrinkage strain in the early stage of RATIC2.

Moreover, as shown in Figure 9b, all curves of RATIC1 were significantly lower than TIC, which indicated a delay in the development of total shrinkage of RATIC1 when RFA1 added. This was due to the fact that the water stored in the RFA1 compensated for the water loss of the cement matrix and contributed significantly to the reduction in the drying shrinkage of the total shrinkage [21]. However, as shown in Figure 9c, RATIC2 did not show a significant delay in the development of total shrinkage. This may be because the long duration of the pozzolanic reaction consumed the water stored in RFA2, so the cement matrix of RATIC2 was unable to replenish water from RFA2.

### 3.4. SEM

**Figure 10:**
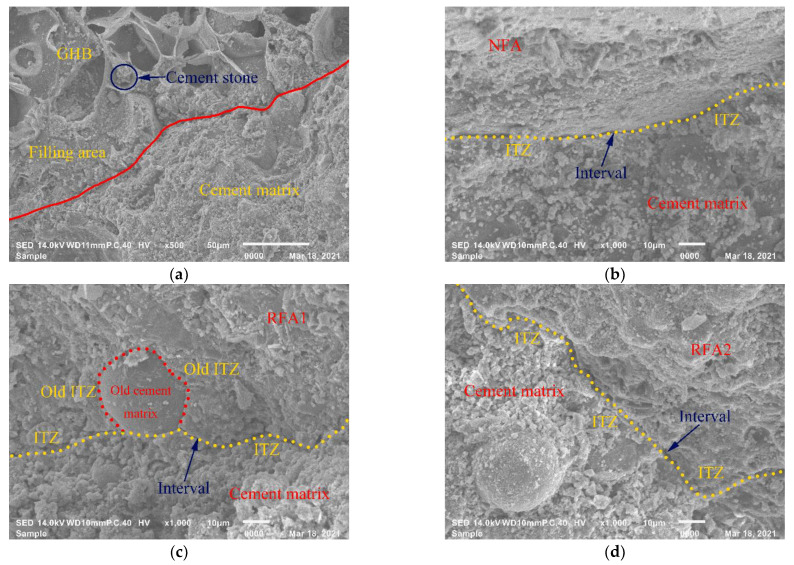
SEM images of (**a**) GHB in TIC (**b**) NFA in TIC, (**c**) RFA1 in RATIC1 and (**d**) RFA2 in RATIC2.

Figure 10a shows the ITZ of TIC between GHB and the cement matrix. There was an obvious filling area between the cement matrix and GHB, and part of the cement matrix filled into the open pores of GHB. Cement matrix filling might be an important reason for the higher total shrinkage strain of TIC than that of NAC in the early stage. It is generally believed that the ITZ was the weak part of concrete, and the analysis of its microstructure was beneficial to further study the performance of concrete. In Figure 10b, there was an obvious ITZ in TIC between NFA and cement matrix. Figure 10c,d show the ITZ of RATIC1 and RATIC2 between fine aggregate and cement matrix, respectively. Compared with TIC in Figure 10b, the ITZ of RATIC1 (Figure 10c) and RATIC2 (Figure 10d) were denser, which explained that the splitting tensile strength and elastic modulus of RATIC1 and RATIC2 could be improved when the RFA replacement ratio was appropriate as shown in Table 6 and Table 7. However, there were also old ITZs between the old natural aggregate and the old cement matrix in RFA1. As shown in Figure 10c, the old ITZ in RFA1 made the ITZ micromorphology more complex, and its length was larger than that of RATIC2, which resulted in worse mechanical properties of RATIC1.

## 4. Time-Dependent Shrinkage Model

The total shrinkage strain (*ε_cs,0_*) considered in the EC2 [41] prediction model is the sum of the autogenous shrinkage strain (*ε_ca_*) and the drying shrinking strain (*ε_cd_*). The specific calculation formulas of *ε_ca_* and *ε_cd_* are shown in Equations (1) and (2).

(1) $$\varepsilon_{ca}=\left(1-e^{-0.2t^{0.5}}\right)\varepsilon_{ca}\left(\infty \right)$$

(2) $$\varepsilon_{cd}=\frac{\left(t-t_{0}\right)}{\left(t-t_{0}\right)+0.04\sqrt{h_{0}{}^{3}}}k_{h}\varepsilon_{cd,0}$$

where *t* is the concrete age, in days; *ε_ca_*(∞) is related to cylinder strength (*f*_ck_) of concrete at 28 days; *t*_0_ is the concrete age, in days, at the beginning of drying shrinkage; *h*_0_ is the nominal thickness of concrete, and 2 times the ratio of the sectional area to the section circumference of the concrete member is taken; *ε*_cd,0_ is the nominal unrestrained drying shrinkage values, which can be found in EN1992 [14]; *k*_h_ is the coefficient depending on the notional size *h*_0_.

The time-dependent shrinkage model for RATIC cites the time-dependent factor *k_wa_* [21], considers the autogenous shrinkage strain amplification factor *k_b_* and drying shrinkage strain amplification factor *k_a_* of RATIC. The specific expression is shown in Equation (3):

(3) $$\varepsilon_{cs}=\left(1-e^{-0.2t^{0.5}}\right)k_{b}\varepsilon_{ca}\left(\infty \right)+\frac{\left(t-t_{0}\right)}{\left(t-t_{0}\right)+0.04k_{wa}\sqrt{h_{0}^{3}}}\gamma_{\alpha }k_{a}\varepsilon_{cd,0}$$

where *k*_b_ is the coefficient depending on the content of GHB; *k*_wa_ and *k_a_* are the coefficients depending on water absorption and elastic modulus of RFA, respectively.

### 4.1. Shrinkage Model for TIC

In the process of concrete solidification and hardening, GHBs inevitably shrink in volume. Therefore, in Equation (5), an amplification factor *k_b_* is introduced to *ε_ca_* to modify the variation of shrinkage strain caused by the volume deformation of GHBs in TIC. By regression analysis of the experimental results of TIC, the autogenous shrinkage strain amplification factor *k_b_* can be calculated. However, GHB is similar to the solid air-entraining agent, which increases the air content of TIC, thus increasing the *ε_cd_* of TIC. According to the volume difference of concrete mixture [8], the volume fraction of GHB is about 25%, which can be considered as the air content intake of TIC and RATIC. The ACI-209-92 [41] model takes into account the influence of concrete air content on the total shrinkage strain of concrete, and gives the correction coefficient *γ_α_*, whose specific value is shown in Equation (4):

(4) $$\gamma_{\alpha }=0.95+0.008\alpha$$

where *γ_α_* is the air content factor, *α* is the air content expressed as a percentage.

The total shrinkage (*ε_cs,b_*) of TIC modified by the autogenous shrinkage strain amplification factor *k_b_* and the air content correction factor *γ_α_* is shown in Equation (5). The autogenous shrinkage strain amplification factor *k_b_* in this experiment was 3.17 by regression analysis.

(5) $$\varepsilon_{cs,b}=\left(1-e^{-0.2t^{0.5}}\right)k_{b}\varepsilon_{ca}\left(\infty \right)+\frac{\left(t-t_{0}\right)}{\left(t-t_{0}\right)+0.04\sqrt{h_{0}^{3}}}\gamma_{\alpha }\varepsilon_{cd,0}$$

**Figure 11:**
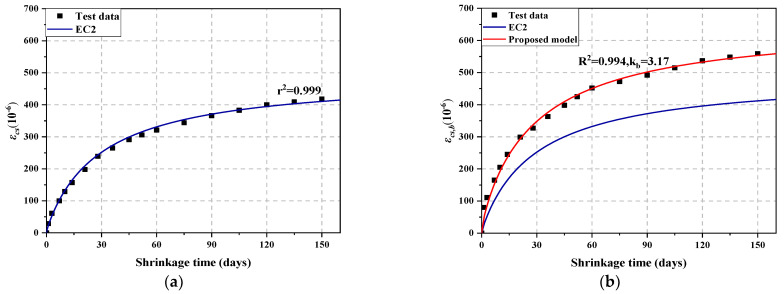
Predicted and tested values of (**a**) NAC and (**b**) TIC.

Figure 11a shows the prediction curve of NAC total shrinkage *ε_cs_* based on the EC2 model, and Figure 11b is the prediction curve of TIC total shrinkage *ε_cs,b_* optimized by the GHB influence factor *k_b_* on the basis of the EC2 model. As shown in Figure 11, the EC2 model can well predict the change trend of the total shrinkage of NAC, but the predicted value of the total shrinkage of TIC differs greatly from the experimental results. However, the model shown in Equation (5) can well predict the variation trend of TIC total shrinkage, indicating that the modification of the EC2 model by introducing *k_b_* is effective.

### 4.2. Time-Dependent Shrinkage Model for RATIC

Researchers found that even if RFA replaces NFA with 100% replacement ratio, the reduction of autogenous shrinkage strain *ε_ca_* of recycled concrete is not more than 20% [17]. Therefore, the autogenous shrinkage model (*ε_ca,b_*) for TIC (Equation (6)) can be used as the RATIC autogenous shrinkage model, and Equation (7) is the TIC drying shrinkage model (*ε_cd,b_*). Due to the delayed effect of RFA on the development of drying shrinkage of RATIC, *ε_cd,b_* cannot predict the drying shrinkage strain of RATIC well. Therefore, the model of *ε_cd,b_* should be modified.

(6) $$\varepsilon_{ca,b}=\left(1-e^{-0.2t^{0.5}}\right)k_{b}\varepsilon_{ca}\left(\infty \right)$$

(7) $$\varepsilon_{cd,b}=\frac{\left(t-t_{0}\right)}{\left(t-t_{0}\right)+0.04\sqrt{h_{0}{}^{3}}}\gamma_{\alpha }\varepsilon_{cd,0}$$

#### 4.2.1. The Citation of Time-Dependent Factor (k_wa_)

The drying shrinkage development coefficient (*β*(*t,t*_0_)) of ordinary concrete in EC2 is shown in Equation (8). In order to describe the experimental phenomenon that the drying shrinkage development time of recycled concrete is longer than that of ordinary concrete, the researchers put forward the time-dependent factor *k_wa_* [21]. The modified drying shrinkage development coefficient (*β_a_*(*t,t_0_*)) is shown in Equations (9)–(12).

(8) $$\beta \left(t,t_{0}\right)=\frac{\left(t-t_{0}\right)}{\left(t-t_{0}\right)+0.04\sqrt{h_{0}^{3}}}$$

(9) $$\beta_{a}\left(t,t_{0}\right)=\frac{\left(t-t_{0}\right)}{\left(t-t_{0}\right)+0.04k_{wa}\sqrt{h_{0}^{3}}}$$

(10) $$k_{wa}=k_{wa-F}k_{wa-C}$$

(11) $$k_{wa-F}=0.10\left[r_{\mathrm{NFA}}\omega_{a-\mathrm{RFA}}+\left(1-r_{\mathrm{RFA}}\right)\omega_{a-\mathrm{NFA}}\right]+2.27$$

(12) $$k_{wa-C}=0.18\omega_{a-\mathrm{NCA}}+0.52$$

where *k_wa-C_* and *k_wa-F_* are the influence coefficients of shrinkage development of coarse aggregate and fine aggregate, respectively; ω*_a-_*_RFA_ is the water absorption of RFA; *r*_RFA_ is the replacement ratio of RFA; *ω_a-_*_NFA_ and *ω_a-_*_NCA_ are the water absorption of NFA and NCA, respectively.

**Figure 12:**
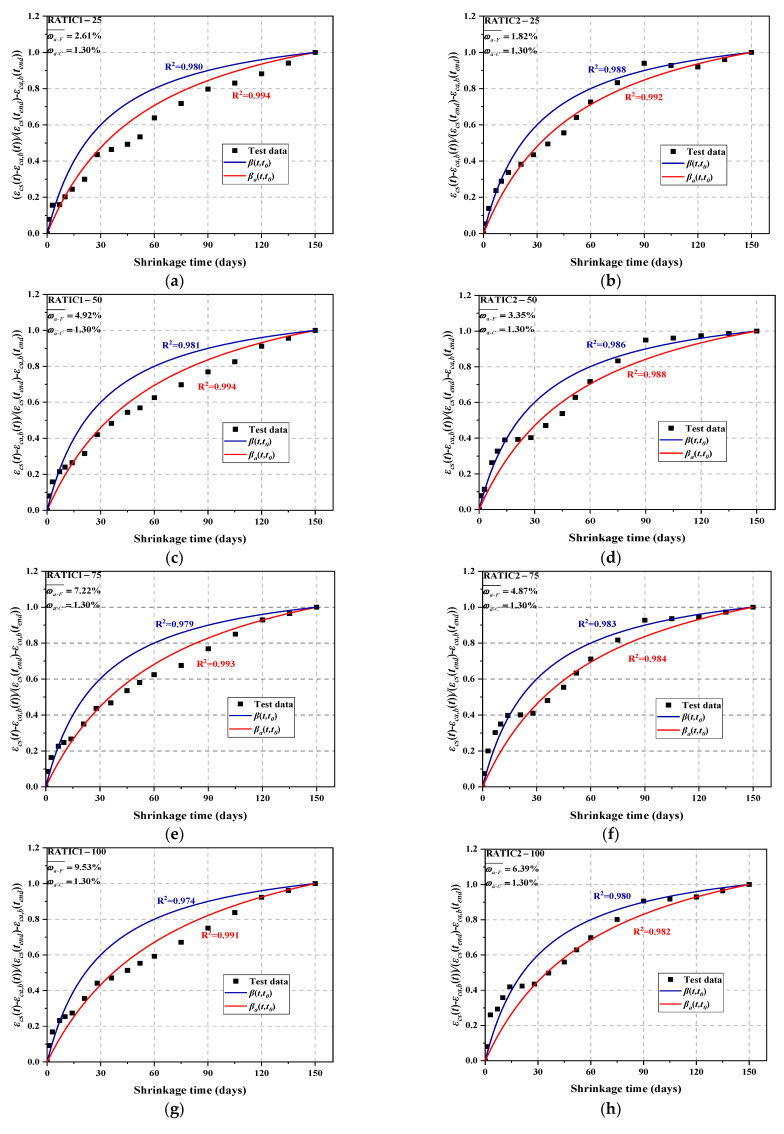
Comparison between the measured and calculated development of normalized total shrinkage with the time of RATIC. (**a**) RATIC1-25; (**b**) RATIC2-25; (**c**) RATIC1-50; (**d**) RATIC2-50; (**e**) RATIC1-75; (**f**) RATIC2-75; (**g**) RATIC1-100; (**h**) RATIC2-100.

In order to express the development of the drying shrinkage strain of RATIC, the difference between the predicted value of total shrinkage strain and autogenous shrinkage strain is normalized, that is, (*ε_cs_*(*t*)-*ε_ca,b_*(*t*))/(*ε_cs_*(*t_end_*)-*ε_ca,b_*(*t_end_*)). *β*(*t,t*_0_) of the EC2 model and the modified *β_a_*(*t,t_0_*) were used to fit the normalized experimental values, as shown in Figure 12a–h. It is observed that the EC2 model with the time-dependent factor *k_wa_* is more helpful to describe the total shrinkage development of RATIC, so it is effective to modify the EC2 model with *k_wa_*.

#### 4.2.2. Drying Shrinkage Strain Amplification Factor k_a_

According to ACI-209R-92 [41], when the quality of cement matrix is the same, the drying shrinkage deformation of ordinary concrete is mainly affected by the volume content of aggregate. The difference in the drying shrinkage between TIC and RATIC mainly lies in the type of fine aggregate. The lower elastic modulus of RFAs increase the *ε_cd_* of RATIC. In order to obtain the drying shrinkage strain model of recycled concrete, researchers usually put forward the drying shrinkage amplification factor *k_a_* based on the drying shrinkage model of ordinary concrete [42]. In summary, time-dependent shrinkage model for RATIC is shown in Equation (3).

**Figure 13:**
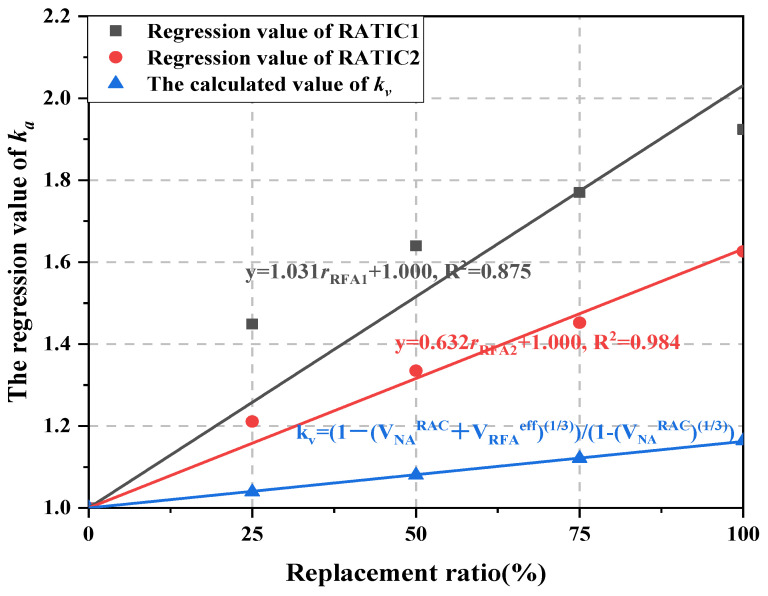
The regression value of *k_a_* and empirical value *k_v_.*

Based on *k_b_* in 4.1, *γ_α_* and *k_wa_* in 4.2.1, Figure 13 shows the RATIC amplification factor *k_a_* obtained by regression analysis of Equation (3) based on test results, *k_v_* is the empirical value of RFAC amplification factor obtained by Zhang et al. [21], as shown in Equation (13). Equations (14) and (15) are the fitting formula of *k_a_* of RATIC1 and RATIC2 with the different replacement ratio of RFA, respectively.

(13) $$k_{v}=\frac{1-{\left(V_{NA}^{RAC}+V_{RFA}^{eff}\right)}^{\frac{1}{3}}}{1-{\left(V_{NA}^{NAC}\right)}^{\frac{1}{3}}}$$

(14) $$k_{a}=1.031r_{RFA1}+1.000$$

(15) $$k_{a}=0.632r_{RFA2}+1.000$$

where $V_{NA}^{RAC}$ and $V_{NA}^{NAC}$ are the volume content of natural aggregate in RAC and NAC, $V_{RFA}^{eff}$ is the effective volume content of RFA providing constraints in concrete, experience value $V_{RFA}^{eff}=0.52V_{RFA}$ [21]. *r*_RFA1_ is the replacement ratio of RFA1; *r*_RFA2_ is the replacement ratio of RFA2.

It can be seen that the regression value of *k_a_* is greater than *k_v_* under the same replacement ratio. This may be because the effective volume of RFA1 and RFA2 are smaller than the experience value of Zhang et al. [21]. At the same replacement ratio, *k_a_* of RATIC1 is greater than that of RATIC2, which also indicates that the elastic modulus of RFA1 is less than that of RFA2.

### 4.3. Validation for the Model

**Figure 14:**
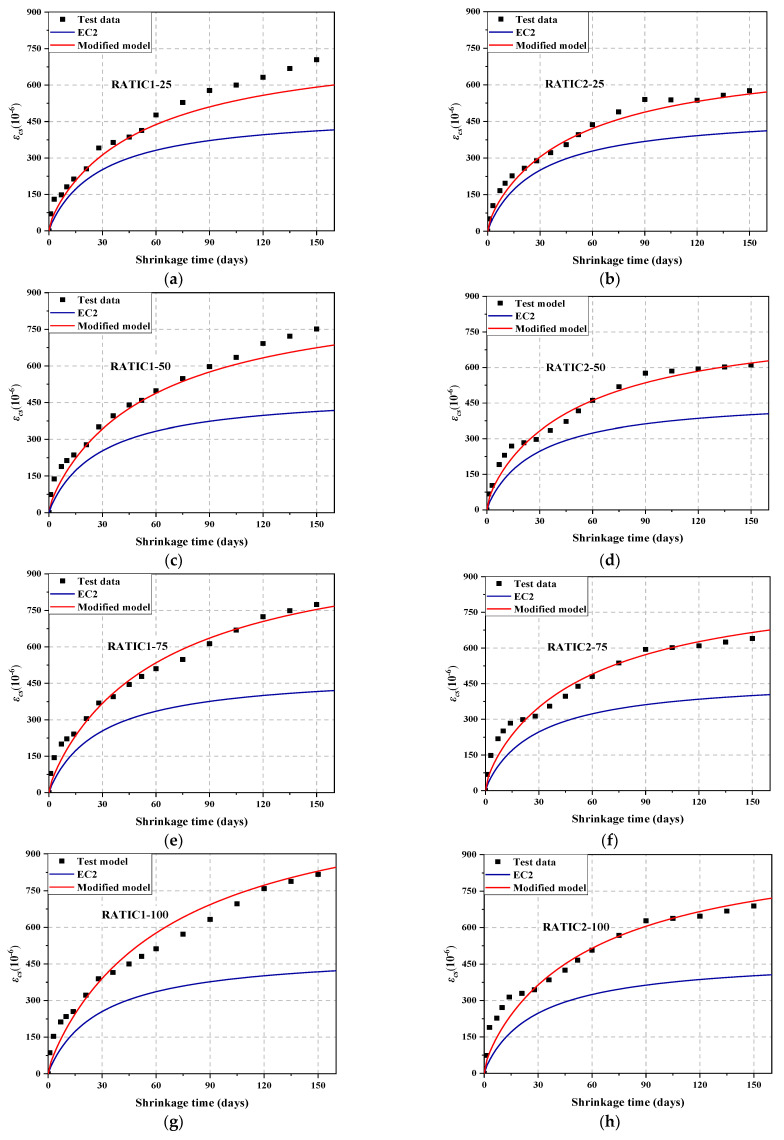
The predicted results of EC2 model and the modified model based on the EC2 model. (**a**) RATIC1-25; (**b**) RATIC2-25; (**c**) RATIC1-50; (**d**) RATIC2-50; (**e**) RATIC1-75; (**f**) RATIC2-75; (**g**) RATIC1-100; (**h**) RATIC2-100.

In order to verify the accuracy of the model, the calculated value of *k*_a_ and *k*_b_ were substituted into Equation (13). Figure 14 shows the predicted results of EC2 model and the modified model based on the EC2 model in this study. It is not difficult to find that the EC2 model optimized by *k_wa_*, *k_b_*, *k_a_*, and *γ_α_* can better predict the change of the total shrinkage of RATIC. It can be observed that the time-dependent shrinkage model can predict both the development trend and final total shrinkage strain of RATIC with a maximum difference of 17%.

## 5. Conclusions

In this experiment, RATIC with GHBs was prepared by using RFA from waste concrete and waste clay brick. The shrinkage performance of RATIC was studied and a time-dependent shrinkage model for RATIC was proposed by considering the effects of GHB and RFA on RATIC shrinkage. The specific conclusions are as follows:

- Adding GHB had no obvious effect on the compressive strength of concrete before 28 days, while decreased the compressive strength after 28 days. In addition, GHB had a negative effect on the splitting tensile strength and elastic modulus of concrete at 28 days.
- The old mortar on the surface of RFA1 weakened the mechanical properties of RATIC1, and the pozzolanic reaction caused by RFA2 effectively improved the mechanical properties of RATIC2. RFA2 was more advantageous to the performance of RATIC than RFA1 in terms of chemical composition. Besides, added RFA decreased the thermal conductivity of TIC.
- The early shrinkage deformation of TIC was significantly greater than that of NAC. With the continuous development of shrinkage, the long-term shrinkage strain of TIC mixed with GHB tended to be stable. The EC2 model modified by the autogenous shrinkage amplification factor kb can effectively predict the TIC shrinkage strain.
- The time-dependent shrinkage of RATIC was similar to that of RFAC due to the influence of water absorption of RFA, and the modified model with time-dependent factor *k_wa_* can effectively predict the drying shrinkage of RATIC.
- The coefficient kb related to GHB content and the correlation coefficient *k_a_* related to the elastic modulus of RFA were introduced to optimize the EC2 model. The time-dependent shrinkage model for RATIC obtained can better predict the total shrinkage strain of RATIC, and the specific expression is shown as Equation (3). In future research, researchers should try to quantify the elastic modulus of different RFAs in order to predict the shrinkage strain of concrete using different RFAs.

## Figures and Tables

**Table 1:** Chemical compositions of cementitious materials (%) [27].

Composition	SiO_2_	Al_2_O_3_	Fe_2_O_3_	TiO_2_	CaO	MgO	K_2_O	N_2_O
Cement	12.54	4.41	3.41	0.59	72.97	0.21	0.90	0.26
Fly ash	38.23	24.76	11.91	1.97	14.85	1.03	1.98	0.61

**Table 2:** Physical properties of GHB.

Size (mm)	Water Absorption (%)	Bulk Density (kg/m^3^)	Thermal Conductivity (W/(m·K))	Cylinder Compressive Strength (kPa)
0.5–1.5	23	130	0.028	209

**Table 3:** Physical properties of natural and recycled aggregate.

Aggregates	Apparent Density (kg/m^3^)	Water Absorption (%)	Moisture Content (%)	Crushing Value (%)	Fineness Modulus
NFA	2650	0.3	0.03	13.20	2.4
RFA1	2330	9.53	4.84	21.88	2.4
RFA2	2350	6.39	1.53	18.41	2.4
NCA	2680	1.30	0.1	14.3	—

**Table 4:** Chemical composition of recycled fine aggregate (%).

	SiO_2_	CaO	Al_2_O_3_	Fe_2_O_3_	MgO	K_2_O	SO_3_	Na_2_O	TiO_2_	BaO	Cl
NFA	96.0	0.50	1.8	0.5	0.1	0.7	0.05	0.20	0.10	0.00	0.00
RFA1	50.52	27.91	11.88	2.09	2.13	1.77	1.33	0.77	0.74	0.26	0.18
RFA2	67.56	1.67	18.23	5.83	1.53	2.22	0.14	1.41	0.94	0.08	0.02

**Table 5:** Mix proportions of RATIC under different replacement ratio (kg).

	Cement	FA ^2^	NFA	RFA	GHB	NCA	Water		SP ^2^	Slump (mm)
							MW ^2^	AW ^2^		
NAC	408	72	475	0	0	1110	240	—	0	180
TIC	408	72	475	0	130	1110	240	—	1.92	190
RATIC1-25 ^1^	408	72	356	104	130	1110	240	4.88	2.88	185
RATIC1-50 ^1^	408	72	238	209	130	1110	240	9.80	3.84	200
RATIC1-75 ^1^	408	72	119	313	130	1110	240	14.68	4.80	205
RATIC1-100 ^1^	408	72	0	418	130	1110	240	19.60	5.76	180
RATIC2-25 ^1^	408	72	356	105	130	1110	240	5.10	2.88	195
RATIC2-50 ^1^	408	72	238	211	130	1110	240	10.25	3.84	205
RATIC2-75 ^1^	408	72	119	316	130	1110	240	15.36	4.80	210
RATIC2-100 ^1^	408	72	0	421	130	1110	240	20.46	5.76	190

^1^ For the nomenclature, taking ‘RATIC1-100′ as an example, RATIC1 stands for recycled aggregate thermal insulation concrete prepared with RFA1; 100 is the replacement ratio of RFA1. ^2^ FA stands for fly ash; MW stands for mixing water; AW stands for additional water; SP stands for superplasticizer.

**Table 6:** Splitting tensile strength of RATIC (MPa).

	NAC	TIC	RATIC1				RATIC2			
*r*_RFA_ (%)			25	50	75	100	25	50	75	100
*f_spt_*	3.73	3.44	3.73	3.81	3.58	2.99	4.51	4.72	4.81	4.25

**Table 7:** Elastic modulus of RATIC (GPa).

	NAC	TIC	RATIC1				RATIC2			
*r*_RFA_(%)	0	0	25	50	75	100	25	50	75	100
*E_c_*	31.4	23.1	23.4	26.7	22.6	15.1	23.8	28.2	30.8	25.9

**Table 8:** Thermal conductivity of RATIC (W/(m·K)).

	NAC	TIC	RATIC1				RATIC2			
*r*_RFA_(%)	0	0	25	50	75	100	25	50	75	100
*Kc*	1.52	0.91	0.85	0.88	0.83	0.81	0.88	0.89	0.90	0.87

## Data Availability

The data used to support the findings of this study are available from the corresponding author upon request.
